# Ideal efficacy photoswitching for chromocontrol of TRPC4/5 channel functions in live tissues

**DOI:** 10.1038/s41589-025-02085-x

**Published:** 2026-01-16

**Authors:** Markus Müller, Konstantin Niemeyer, Navin K. Ojha, Sebastian A. Porav, Deivanayagabarathy Vinayagam, Nicole Urban, Fanny Büchau, Katharina Oleinikov, Mazen Makke, Claudia C. Bauer, Aidan V. Johnson, Stephen P. Muench, Frank Zufall, Dieter Bruns, Yvonne Schwarz, Stefan Raunser, Trese Leinders-Zufall, Robin S. Bon, Michael Schaefer, Oliver Thorn-Seshold

**Affiliations:** 1 Faculty of Chemistry and Food Chemistry, Dresden University of Technology, Dresden, Germany; 2 Department of Pharmacy, Ludwig-Maximilians University of Munich, Munich, Germany; 3 Rudolf-Boehm-Institute of Pharmacology and Toxicology, Leipzig University, Leipzig, Germany; 4 Center for Integrative Physiology and Molecular Medicine, Saarland University, Saarbrücken, Germany; 5 Leeds Institute of Cardiovascular and Metabolic Medicine, LIGHT Laboratories, University of Leeds, Leeds, UK; 6 Astbury Centre for Structural Molecular Biology, University of Leeds, Leeds, UK; 7 Department of Structural Biochemistry, Max Planck Institute of Molecular Physiology, Dortmund, Germany; 8 School of Biomedical Sciences, University of Leeds, Leeds, UK

**Keywords:** Structural biology, Transient receptor potential channels, Chemical tools, Molecular neuroscience

## Abstract

Precisely probing the endogenous roles of target proteins is crucial for biological research. Photochemical tools can be photoactuated with high spatiotemporal resolution but often they are unreliable in vivo because spatiotemporal variations of reagent concentration result in inhomogeneous bioactivity. We now describe ideal efficacy photoswitching, a paradigm that internally compensates for reagent concentration by self-competitive binding, allowing purely wavelength-dependent chromocontrol over bioactivity that is consistent from cell culture to deep tissues. We demonstrate this with photoswitches for endogenous transient receptor potential (TRP) C4 and C5 ion channels, reproducibly delivering strong agonism under 360-nm illumination, weak agonism under 385-nm illumination and strong antagonism under 440-nm illumination. These ligands unlock a range of high-precision investigations in TRP biology, from neuronal activity to exocytosis, reproductive signaling and smooth muscle contractility. The ideal efficacy photoswitching paradigm should also unlock high-performance chromocontrol over a wide range of sensory or signaling channels and receptors even in vivo.

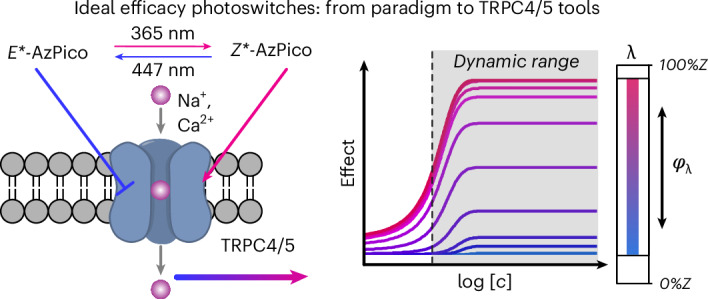

## Abstract (web summary)

Using chemical photoswitchable reagents to exert purely wavelength-dependent control over biological systems in deep tissue and in vivo requires a concentration-independent design paradigm. Here, such photoswitchable ligands are realized by ensuring that *E*/*Z* isomers have opposing efficacies yet similarly high affinity, allowing them to probe transient receptor potential C4 and C5 channel functions up to the tissue level.

## Main

The 27 human transient receptor potential (TRP) proteins can assemble to form tetrameric TRP cation channels, which have varied and highly tissue-dependent roles in cellular physiology, from sensing and signaling to mineralostasis^1,2^. Several environment-sensing TRP channels such as TRPV1 (heat), TRPM8 (cold) or TRPA1 (electrophiles) are well studied. The roles and importance of many other TRP channels remain unclear, even though their links with diseases make them hotly pursued as therapeutic targets^3–5^. The structurally similar TRPC4 and TRPC5 (TRPC4/5) are mainly expressed in the central nervous system, gut and kidneys^6–8^, where they are linked with pain^9–11^, reproductive signaling^12^, anxiety and depression^13,14^, kidney disease^15^ and digestion^16^; striking discoveries are still ongoing, for example, linking TRPC5 loss to postpartum depression and obesity^17^. TRPC4/5 form both homotetrameric and heterotetrameric functional channels that can also include TRPC1 (ref. ^18^).

**Fig. 1:**
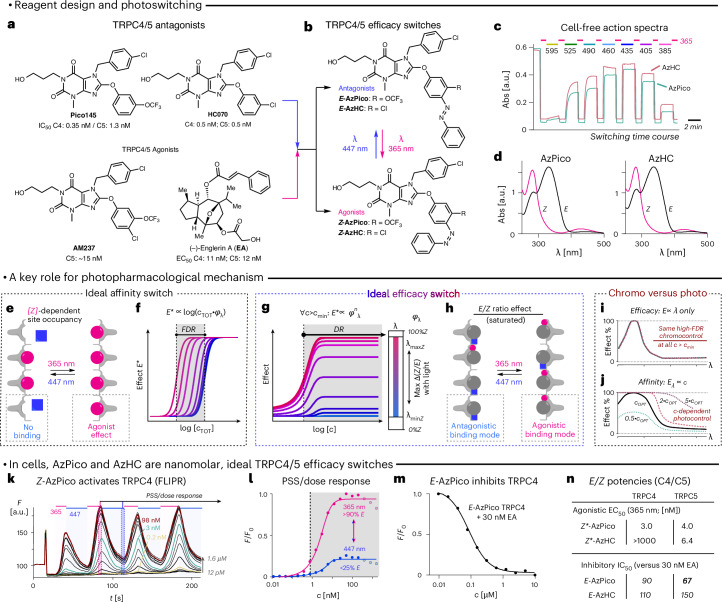
Ideal efficacy photoswitches for TRPC4/5. **a**, Known TRPC4/5 modulators. **b**, Photoswitchable TRPC4/5 modulators AzPico and AzHC. **c**,**d**, Photoisomerization action spectra and *E*/*Z* isomer absorption spectra (derived for individual isomers from high-performance liquid chromatography) of AzPico and AzHC. **e**,**f**, For an ideal affinity switch, only one isomer binds the target. Within the FDR window, the binding site occupancy and, thus, biological effect E* depend on the total switch concentration *c*_TOT_ and the PSS fraction of active isomer φ_λ_. **g**,**h**, For an ideal efficacy switch, both isomers bind with similar affinities but with different efficacies. The dynamic range (DR) where the biological effect E*_λ_ is PSS dependent but concentration independent covers all *c* > *c*_min_. **i**,**j**, Chromocontrol in practice: for an efficacy switch, small variations in PSS(λ) sensitively control performance (whereas, in affinity switches, they are unimportant) but even large variations in concentration, which would ruin the performance of an affinity switch, are irrelevant. **k**,**l**, Reversible Ca^2+^ influx modulation with AzPico under 365/447-nm cycles, as time courses and peak amplitudes. **m**, *E*-AzPico binds competitively to EA. **n**, EC_50_ and half-maximal inhibitory concentration values of *E/Z*-AzPico and AzHC on TRPC4 and TRPC5. **k**–**n**, Fluo-4-loaded HEK_mTRPC4β_ cells. Source data

Insights into TRPC4/5 relevance were initially derived from knockout mice because potent and selective modulators were lacking^19^. Recently, small-molecule modulators of TRPC4/5 became available as tool compounds^20^: from the nanomolar-potent natural product (−)-englerin A (EA)^21^, a TRPC1/4/5 activator^22,23^, to drug candidate inhibitors (for example, TRPC4/5-targeting xanthines BI-1358894, HC-070 and Pico145/HC-608 developed by Hydra/Boehringer or TRPC5-targeting pyridazinones GFB-8438 and GFB-887 developed by GoldfinchBio) (Fig. 1a and Supplementary Note 1)^24,25^. However, TRP functionality varies according to tissue localization (spatial) and TRPs are best studied if their activity is reversibly modulated on short timescales (temporal). Therefore, photoresponsive chemical tools, whose activity can be spatiotemporally patterned by light, are particularly promising to resolve TRP biology^26^. Photoswitchable TRP ligands have been impactful, with azo-capsaicins for TRPV1 (refs. ^27,28^), TRPswitch for TRPA1 (ref. ^29^), OptoBI for TRPC3 (ref. ^30^) and azo-diacylglycerols (PhoDAGs and OptoDArGs) for TRPC2/3/6 (refs. ^31–33^). However, there is no photoresponsive ligand for TRPC4 and the sole compound for Trpc5 (BTDAzo, a lipophilic photoswitchable agonist) has low potency and is almost inactive on human TRPC5 (ref. ^34^).

Here we sought to create potent, selective, photoswitchable TRPC4/5 modulators for spatiotemporally resolved TRP studies in complex systems. Xanthines such as HC-070 and Pico145 were our choice for parent ligands, as small chemical changes switch them from activators to inhibitors^35,36^, in turn suggesting that a compound could be designed such that photoswitching it would likewise change its efficacy. We now report the design and experimental validation of this concept and use these tools for light-controlled high-precision elucidation of the separate roles of TRPC4 and TRPC5 from cell culture through to endogenous organ sections; as this efficacy switch paradigm solves systematic problems that have hampered the field of photopharmacology for decades, we argue for adopting it more broadly as a method to unlock high-precision in vivo chromocontrol of a range of biological systems.

## Results

### Design concept for efficacy switching

Azobenzenes are the best-explored chemical scaffolds for fully reversible structural photoswitching (through *E*⇆*Z* isomerization)^37^ and we decided to use them in ligands to photoswitch TRPC4/5 bioactivity. However, azobenzene *E*⇆*Z* photoswitching is never complete in both directions: it operates between mostly *Z* and mostly *E* photostationary states (PSSs, typically ~90% *Z* for the mostly *Z*-PSS [Z*] and ~80% *E* for the mostly *E*-PSS [E*]).

Affinity switching is the typical pharmacological approach used for structure–activity relationship-based photopharmacology; for example, differences in the steric fit of *E* and *Z* isomers drive a difference in their binding affinity, such that net *E*⇆*Z* photoswitching modulates the target’s biological activity. One well-known problem, even for a hypothetical ‘ideal’ affinity switch (with a completely nonbinding *E* isomer but a high-agonist-potency *Z* isomer; Fig. 1e), is the high background activity after typical *Z* → *E* photoswitching ‘off’ because of the typically 20% residual *Z* isomer in the mostly *E*-PSS. The generalized result is that bidirectional photocontrol of an affinity switch can only deliver a narrow functional dynamic range of bioactivity (FDR)^38,39^, that is, the bioactivity window between the best photoswitched on and best photoswitched off states (Fig. 1f; the FDR is limited by its PSSs in both directions). In theory, this could be remedied by making affinity switches with bidirectionally complete photoswitching. Yet, there is a second systematic problem, which has long been ignored and cannot be fixed no matter how complete the photoswitching or, if fast *Z* → *E* relaxation is used, how extreme the affinity difference or how red-shifted the wavelengths. This problem is that the bioactivity applied under any given wavelength remains extremely sensitive to concentration (Fig. 1j), such that both dose and wavelength must be dynamically balanced to deliver a given effect. We suggest that this explains why the applicability of freely diffusing affinity switches for bidirectional photocontrol has been and will remain limited to highly controlled cell culture settings^40^ where their concentration is effectively clamped (Supplementary Note 2). This is because, in tissue or in vivo, spatially variable reagent concentrations that also evolve heterogeneously over time (absorption, distribution, metabolism and excretion, pharmacokinetics, distance from blood vessels and interanimal variability) will prevent delivering a reliable, homogeneous effect.

Efficacy switching is the pharmacological^41^ approach we use here, although we will have to newly define an ‘ideal’ class of efficacy photoswitch, with importantly distinct properties, to reach its potential. An efficacy photoswitch is a reagent whose *E*/*Z* photoisomers exert different efficacies on a target. Unhelpfully, this covers reagents of limited practical utility, for example, whose *E*/*Z* isomers have similar degrees of the same efficacy (for example, 60% versus 70% partial agonism), such that biologically applicable photocontrol is impossible, or whose *E*/*Z* isomers have very different affinity (typically ≥10×), such that, in practice, they act as affinity switches with their associated problems and/or whose *E*/*Z* isomers have such low affinity (for example, half-maximal effective concentration (EC_50_) > 1 µM) that the reagent cannot be reliably applied at saturation (>10 × EC_50_) and, thus, cannot overcome concentration inhomogeneities. Any one of these problems makes an efficacy photoswitch nonideal.

We, therefore, define an ideal efficacy photoswitch as a reagent whose *E*/*Z* isomers bind competitively to each other, with identical or very similar affinity, whose *E*/*Z* isomers have opposing activity, for example, activator/inhibitor (or else one isomer is a silent binder) (Fig. 1h), and where both *E*/*Z* isomers have high potency such that plateau concentrations of 10–100 × EC_50_ can be reliable applied (for example, both EC_50_ < 50 nM). The novelty of creating such a nanomolar ideal efficacy switch on TRPC4/5-binding scaffolds is the chemical highlight of this paper and is the key to their biological utility. First however, it will be crucial to trace two advantages of ideal efficacy switching, as the pharmacological issue of how a compound exerts its photocontrol has not yet received proper formal attention (especially compared to, for example, how much literature is devoted to easily measured but less critical aspects such as incremental tuning of photoresponse wavelengths and PSSs).

1. Chromocontrol is biologically practical: For an ideal efficacy switch, above a threshold for the total *E*/*Z* concentration where its target nears saturation by any *E*/*Z* mixture (~5 × EC_50_), there is no more concentration dependency of bioactivity. The only variable controlling bioactivity is the *E*/*Z* ratio of the competitively binding isomers: that is, the applied color (wavelength) of light sets the PSS, which entirely controls the bioactivity on the target. Just shifting the wavelength can rheostat the target to, for example, strongly on, weakly on, baseline or strongly inhibited states. We now specifically term this target ‘chromocontrol’, from the Greek *chromos* (color); this distinguishes it from ‘photocontrol’ that is used to indicate a general response to light often with affinity switches, but never with the implication of concentration-independent chromocontrol (Fig. 1g,i). Reproducibly and homogeneously applying a specific wavelength of light is far easier than trying to achieve reproducible, homogeneous drug concentrations even across in vitro models, let alone in vivo, suiting efficacy switches to easy translation between model systems and making them ideal or even irreplaceable to tackle complex environments.
2. Chromocontrol is biologically meaningful: Only plateau regions in dose–response curves can be used robustly when concentrations are variable. As an efficacy photoswitch’s bioactivity is equally controlled by wavelength at any concentration above its threshold, the working concentration window within which a biological effect is (identically) PSS dependent is vast (Fig. 1g,i). Furthermore, even a hypothetical ideal affinity switch only has two plateau effect values (full on/full off; Fig. 1f); hence, when concentrations are variable, the switch can only reliably be applied for full on and full off photocontrol. By contrast, ideal efficacy switches, with wavelength-dependent plateaus across their full effect range (for example, strongly on, weakly on, baseline and strongly inhibited), allow reliable rheostatting of bioactivity, which can be more nuanced and biologically meaningful (for example Fig. 6g,h).

These features should make efficacy photoswitches much better suited for robust and sensitive use in biology, from cell culture through to in vivo (Figs. 3–6). However, only a dozen cases of efficacy photoswitching have been published openly (chemokine, adenosine, cannabinoid, adrenergic and serotonin receptors). Key contributions include those of Leurs, Decker, Gorostiza and coworkers (details in Supplementary Note 2)^42–53^. However, these all class as nonideal, typically because (1) the ligand efficacies were switched between more and less activating, rather than, for example, activating and inhibiting and (2) the isomers’ affinity was very different, which compromised the concentration independence of ideal efficacy switching. The closest to ideality was the Leurs chemokine photoswitch VUF16216 (refs. ^43,48^); however, it had micromolar activity that prevented it from exploiting effect plateaus for chromocontrol. Several other efficacy switches have been created but were not understood as such. For example, we published one reagent^34^ whose excellent bioactivity photocontrol despite *E*⇆*Z* photoswitching incompleteness (a hallmark of efficacy switching) we failed to understand. Moreover, we believe that many more instances of unsuspected efficacy switches may be found by reparsing the literature (Supplementary Note 2 has examples from Fuchter, Groschner, Pepperberg, Trauner, etc.^29,30,54–56^).

We give this level of detail as we believe that ideal efficacy photoswitching is both necessary and sufficient to reach the naively popular picture of ‘light control over biology’, which motivated much of the photopharmacology over the last decades. We also did not find it collected accessibly in the literature elsewhere. However, the conceptual importance of this framework goes deeper than pharmacology. As one example, we highlight that there are target-driven reasons to choose efficacy switching for proteins that are natively poised for steeply nonlinear dose–response switching between metastable states, such as receptors and ion channels with multiple binding sites. These ought to be ideal platforms where the competitive binding of similar-affinity *E/Z* isomers with opposing modes of action ought to allow concentration-independent, rheostatted chromocontrol over protein activity, even when *E*⇆*Z* photoswitching is incomplete (see Discussion). A separate theoretical paper will treat these aspects in detail; however, for now, we set out to test this concept in practice, by creating such ‘ideal efficacy switch’ reagents for TRPC4/5.

### Creating xanthine efficacy switches

Xanthines Pico145 (also called HC-608) and HC-070 (ref. ^57^) are TRPC1/4/5 antagonists with picomolar potency^58^ and remarkable selectivity against hundreds of enzymes, receptors, transporters and other ion channels (including other TRP channels)^24^. Excitingly, the very similar AM237 (ref. ^57^) is instead a nanomolar agonist of homotetrameric TRPC5, despite also being a nanomolar antagonist of homotetrameric TRPC4 (full details of pharmacology in Supplementary Note 1)^35^. The structures of these compounds with different efficacy are nearly identical (Pico145: *m-*OCF_3_, HC-070: *m-*Cl; AM237: *m*-OCF_3_, *p*-Cl; Fig. 1a). This suggests that the *meta*/*para* positions are suitable as an ‘ideal efficacy tipping point’, whereby small modifications may flip the efficacy mode (activator or inhibitor) without changing the binding affinity. Overall, the xanthines seem to be an outstanding starting point for ideal efficacy switches that are also highly potent; thus, they can be reliably applied in vivo.

In brief, we synthesized a series of xanthines ‘extended’ with bidirectionally switchable azobenzenes (Supplementary Fig. 3). Noteworthily, with a simple NNPh motif in *para* (where AM237 has a -Cl), we obtained AzPico (*m-*OCF_3_) and AzHC (*m-*Cl; Fig. 1b), which were soon identified as the most biologically useful candidates in our panel of eight. From here onward, we focus only on them, leaving the others to Supplementary Note 3. AzPico/AzHC could be reversibly photoswitched between PSSs of ~82%*E* around 410 nm and 95%*Z* around 360 nm (Fig. 1c,d, and Supplementary Table 3).

### Parallel-throughput chromocontrol assessment in cells by FLIPR

We initially screened for the photoswitchability of activity in cells using a fluorometric imaging plate reader (FLIPR) calcium flux assay, with HEK293 cells stably expressing mouse TRPC4β or mouse TRPC5. Note, however, that the parallel-throughput FLIPR setup is limited to use fixed light-emitting diodes at 365 nm (good *Z*) and 447 nm (suboptimal *E*), which, combined with the slowness of the onset and recovery of the fluorescence readout for calcium, causes the FLIPR results to underestimate the reagents’ true speed, degree and completion of TRPC chromocontrol (shown later).

*E-*AzPico from 12 pM to 1.6 µM gave no effect with TRPC4 or TRPC5; upon 365-nm illumination, strong agonism was evident with Ca^2+^ influx rapidly evoked at low-nanomolar concentrations and this was rapidly photoreversible with 447-nm illumination, over many cycles (Fig. 1k). The remarkable observation that the maximum and minimum calcium signals are dose independent over a concentration range of >100-fold (from nanomolar to micromolar), even though the concentration of the agonistic *Z* isomer likewise increases >100-fold in this range, confirms it as an ideal efficacy switch (TRPC4 in Fig. 1g, l; TRPC5 in Supplementary Fig. 2 (*E*) and Supplementary Fig. 13 (*Z*)). Pleasingly, it inherits the high potency of its parent Pico145, with a 365-nm EC_50_ of just 3.0 nM on TRPC4 and 4.0 nM on TRPC5 (Fig. 1n). AzHC was also a *Z-*agonistic ideal efficacy switch for TRPC5 with a 365-nm EC_50_ of 6.4 nM. Excitingly, however, *Z-*AzHC was completely inactive on TRPC4 into the micromolar range (Supplementary Fig. 13). Competition assays supported that these compounds are indeed efficacy switches (Fig. 1m and Supplementary Note 1).

Taken together, these reagents are uniquely potent efficacy switches; AzPico is a photoswitchable tool addressing TRPC4; although AzPico acts on TRPC5 as well, the cellular role of TRPC4 can be tested by applying it comparatively to the TRPC5-selective AzHC, making them an outstanding reagent pair for probing these otherwise hard-to-resolve channels. Retaining the nanomolar potency of their optimized parent compounds is also a rarity among photopharmaceuticals because the extra moiety needed for photoisomerization usually sacrifices potency.

### Photomodulated electrophysiology

We next performed electrophysiological patch clamp experiments to characterize the action and specificity of channel modulation in more detail. Although its throughput is lower, electrophysiology is more powerful than FLIPR in several respects. Firstly, the nonoptical electrophysiology readout does not cause unwanted photoswitching, unlike FLIPR. Secondly, electrophysiology readouts linearly and temporally resolve ionic currents through the activated channels, unlike the delayed and attenuated fluorometric Ca^2+^ influx analyses in FLIPR. Thirdly, the electrophysiology setup can use narrow-bandwidth monochromated light at any wavelength. These features allow measuring the full power of the photoswitch reagents.

**Fig. 2:**
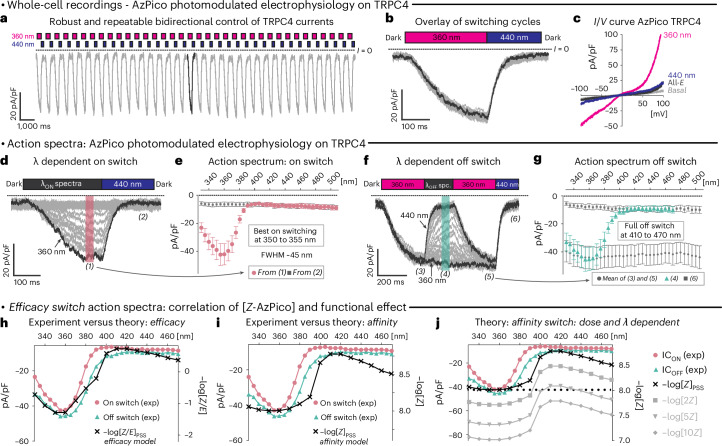
AzPico-chromocontrolled electrophysiology of TRPC4. **a**–**g**, Electrophysiological whole-cell recordings of TRPC4 currents in voltage clamp mode (**a**,**b**,**d**–**g**, *V*_h_ = −80 mV; **c**, *V*_h_ scan) in HEK293 cells with 10 nM AzPico during photoswitching. **a**,**b**, Reproducibility of 36 consecutive photoswitching cycles of 360/440 nm (**a**, time course; **b**, overlay of all cycles). **c**, *I*/*V* curves showing that 440 nm drives almost a full return to baseline currents throughout the applied voltage range. **d**–**g**, Spectral scans to extract the wavelength dependency of channel current photoswitching on (**d**,**e**, cycles of λ_ON_/440 nm) and photoswitching off (**f**,**g**, 360 nm/λ_OFF_). In **e**,**g**, *n* = 12 biological replicates; data are presented as the mean values ± s.e.m. **h**–**j**, Electrophysiology action spectra of AzPico match PSS-based expectations for an efficacy switch (**h**) but not an affinity switch (**i**), which is an important distinction, as an affinity switch would have severely concentration-dependent activity (**j**) (full legend in Supplementary Information). Source data

We thus recorded wavelength-dependent action spectra of AzPico and AzHC under fast photoswitching in electrophysiology in TRPC4/5-expressing cells (Fig. 2 and Supplementary Fig. 14), higlighting the potential of ideal efficacy switches. For example, AzPico reversibly photomodulated TRPC4 currents over >36 consecutive cycles with a fully constant activation profile, without fatigue (Fig. 2a,b). *I*/*V* curves showed a strong activation of ion flux at best *Z* (360 nm) PSS; yet, only at nonphysiological voltages (<−80 mV or >+60 mV) could any small differences between basal activity and good *E* (440 nm) PSS be detected (Fig. 2c), making them truly off–on TRPC modulators. Reversal potentials were close to 0 mV, indicating no gross changes in the permeabilities of monovalent versus divalent cations.

As native TRPC4/5-bearing TRPC complexes are mostly heteromeric TRPC1:TRPC4 or TRPC1:TRPC5 assemblies, we tested AzPico and AzHC in cells that coexpress TRPC1 with TRPC4/5 and found photoresponse *I*/*V* curves typical for the heteromeric complexes (Supplementary Fig. 15). This supports that AzPico and AzHC give comparable chromocontrol over the TRPC1-containing heteromeric TRPC complexes to that over homomeric channels. This is noteworthy, as AM237, for example, acts strictly on TRPC5 and does not activate heteromeric TRPC1:TRPC5 (ref. ^35^). This flexibility favors the use of AzPico and AzHC as versatile reagents for endogenous settings (tested in Figs. 4–6).

The repeatability of on–off photocycling allowed us to extract action spectra for both (1) photoactivation (Fig. 2d,e) and (2) photodeactivation (Fig. 2f,g) in situ in live cells. Channel currents were optimally photoswitched on in a sharp wavelength range of 340–370 nm and full photoswitching off was triggered over the broad range of 400–480 nm (best: 430 nm). Such action spectra conclusively matched the highly wavelength-sensitive model expected from cell-free PSS measurements for an ideal efficacy switch (bioactivity ∝ −log_10_([*Z*]/[*E*])) but mismatched the model for an affinity switch (bioactivity ∝ −log_10_([*Z*])); this (mis)match was visible even for single-concentration data (Fig. 2h,i).

Importantly, we recall that only the efficacy switch mechanism allows these reagents to deliver binary off–on bioactivity that is identically wavelength dependent at any concentration (Fig. 1g–i and Fig. 2h); for example, if they were classical affinity switches, even doubling their concentration would prevent photoswitching-off cell currents (Fig. 2j and Supplementary Note 1). Thus, the concentration-scan, fixed-wavelength FLIPR and the fixed-concentration, wavelength-scan electrophysiology (Fig. 1k,l, Fig. 2e,g,h and Supplementary Fig. 14) show that AzHC and AzPico are ideal efficacy switches, whose *E*/*Z-*competitive binding controls TRPC4/5 currents with exquisite wavelength sensitivity and concentration independence.

### Paired pairs of cryo-electron microscopy TRPC4/5:*E*/*Z* structures indicate activation mechanism

Single-particle cryo-electron microscopy (cryo-EM) studies of TRPC4 (refs. ^59–61^) and TRPC5 (refs. ^62–65^) have given important insights into the structures of these channels and their complexes with lipids, metals, proteins and small-molecule modulators. These include TRPC5 structures with Pico145 (ref. ^64^) and HC-070 (ref. ^62^), binding in near-identical pose to the same lipid-binding pocket, adjacent to the pore helices. However, no channel-open structures are known and all reported ligand structures are with inhibitors^36^. As AzHC and AzPico should bind with high affinity in both *E* and *Z* forms, this offered an opportunity to elucidate the structural basis of inhibitory versus activating efficacy on TRPC4/5, as well as the remarkable differential of TRPC4 activity induced by the -Cl/-OCF_3_ swap. *E*/*Z* structure pairs could also be valuable for rational photopharmacology, as only two protein–ligand structure pairs with an azobenzene reagent bound as both *E* and *Z* isomers^49,66^ are known to date.

**Fig. 3:**
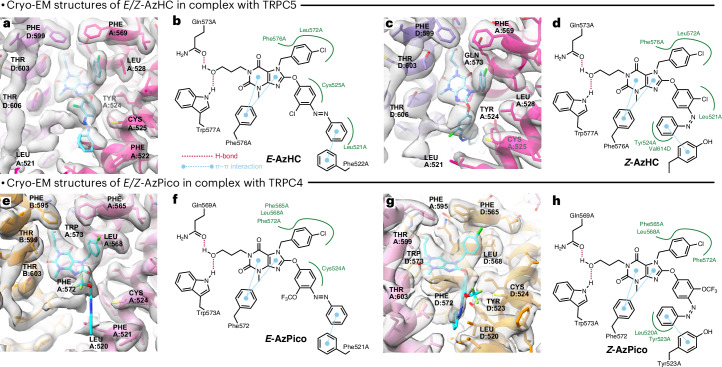
Structures of TRPC4/5 in complex with *E*/*Z* isomers of efficacy photoswitches. **a**–**d**, hTRPC5:*E*/*Z*-AzHC complexes (*E:* 2.6 Å, PDB 9G4Y, EMD-51074; *Z:* 2.9 Å, PDB 9G50, EMD-51076; *C*_1_ symmetry). Note the flip of the distal azobenzene ring. **e**–**h**, TRPC4_DR_:*E*/*Z*-AzPico complexes (*E:* 3.0 Å, PDB 9FXL, EMD-50850; *Z:* 3.1 Å, PDB 9FXM, EMD-50851; *C*_1_ symmetry). Each complex’s best-resolved xanthine-binding site is highlighted. Two-dimensional maps of ligand–protein interactions are shown.

We now studied TRPC5:*E*/*Z-*AzHC and TRPC4:*E*/*Z-*AzPico complexes by cryo-EM, hoping to acquire the ‘paired pair’ of both inhibited and activated structures for both channels. This work was run fully independently for TRPC4 at one site and TRPC5 at another, yet the results aligned, giving confidence in their interpretations. We could determine all four structures at high resolution (human (h)TRPC5:*E*-AzHC, 2.6 Å; hTRPC5:*Z*-AzHC, 2.9 Å; TRPC4_DR (*Daniorerio*)_:*E*-AzPico, 3.0 Å; TRPC4_DR_:*Z*-AzPico, 3.1 Å) without imposing symmetry during data processing (Fig. 3, Supplementary Figs. 16–20, Supplementary Tables 4–7 and Supplementary Videos 1–4). We used 365 nm for *Z* ligand structures and dark or 440 nm for *E* structures; furthermore, we excluded DTT from all buffers to avoid diazene reduction.

Supplementary Note 4 contains full details of the structural biology results. Briefly, *E*/*Z-*AzHC/AzPico could be built into the expected lipid-binding or xanthine-binding site, with near-identical positions to that of Pico145 (ref. ^64^) for the conserved ligand portion and differences between the *E* and *Z* isomers only at the azobenzene. For example, the distal ring of *E*-AzHC is projected outward to make a *π*–*π* interaction with Phe522 of TRPC5, whereas, in *Z*-AzHC, it folds deeply inward to make a *π*–*π* interaction with Y524; AzPico behaves similarly on TRPC4 (Fig. 3 and Supplementary Figs. 4–6). Most protein residues in the ligand-binding site are in similar positions in the *E*/*Z* structures; the exceptions are that, for hTRPC5, Phe520 is flipped ‘in’ or ‘out’ depending on whether the antagonistic *E* or agonistic *Z* isomer is bound (Supplementary Fig. 4b), while, in TRPC4_DR_, the cognate Phe521 is much less shifted, although its neighboring L520 is notably displaced (Supplementary Videos 1–4). All *E* and *Z* structures had the channel pore closed (potential reasons for this in Supplementary Figs. 5–7); thus, caution in interpreting the agonist structures is needed. Nevertheless, these data offer structural insight into how closely related xanthines can have opposite effects on TRPC4/5 function (that is, inhibition versus activation).

With these binding modes confirmed, we tested whether the selective activation of TRPC5 but not TRPC4 by *Z-*AzHC could be the result of the single-amino-acid difference in their binding sites (Val579 in TRPC5 versus Ile575 in TRPC4 at the cognate position). However, neither the Val579Ile nor the Ile575Val substitution in TRPC5 and TRPC4, respectively, changed their activity profiles for *E*/*Z-*AzHC or for control activator AM237. This suggests that the basis for *Z-*AzHC’s TRPC5-selective activation is more complex than the immediate residues it contacts (Supplementary Note 4).

### Photoswitching endogenous TRPC4/5 in primary cells to photoreversibly actuate cell function

**Fig. 4:**
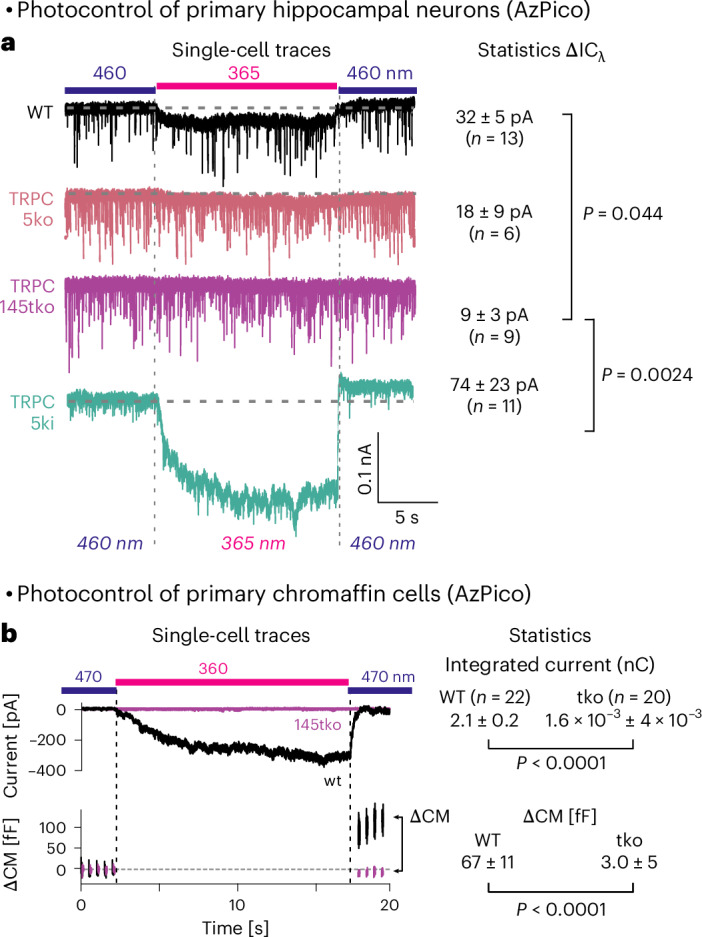
AzPico (30 nM) photoswitchably evokes currents in primary neuronal and neuroendocrine cells. **a**, Photoswitching-based current differentials ΔIC_λ_ in hippocampal neurons (currents at 365 nm relative to 460 nm). **b**, Photoswitch-based currents and net charge transfer (top) correlate with ΔCM (bottom), indicating that phototriggering of TRPC[1]/4/5 leads to exocytosis (details in Supplementary Fig. 21). Left, single-cell traces; right, group statistics. Source data

We next moved to test whether AzPico can directly chromocontrol endogenous TRPC4/5, using autaptic hippocampal neurons (neurons cultured in isolation, which only make synapses back onto themselves, as a model for simultaneously monitoring presynaptic and postsynaptic responses). The 365/460-nm cycles reversibly activated inward currents in wild-type (WT) neurons, just as was seen in heterologously TRPC4/5-(over)expressing HEK cells (Fig. 2). These WT neurons consist of a fraction of cells expressing TRPC channels that can be directly activated by AzPico plus about 50% of neurons that do not express TRPC channels^67^. Matching expectations, average currents were larger when acquired exclusively from TRPC5-bearing cells (5ki), strongly depressed with TRPC5 single knockout (5ko) and almost abolished with TRPC1/C4/C5 triple knockout (145tko), supporting that TRPC[1]4/5 channels are the central contributor to AzPico’s photomodulation of membrane conductance (Fig. 4a)^68^. This shows that AzPico can optically control the activity of native TRPC[1/4/]5 channels in primary nerve cells without channel overexpression.

We then tested the photopharmacology of AzPico in isolated primary chromaffin cells (the neuroendocrine cells in the adrenal gland that secrete adrenaline by exocytosis in response to electrical activation and functionally express TRPC1/4/5 channels)^69^. The 360-nm illumination of AzPico photoreversibly triggered robust inward currents that caused an increase in membrane capacitance (CM) in WT cells, indicative of exocytosis (Fig. 4b). Neither response was observed in 145tko cells, confirming the TRPC specificity of this photopharmacology. By supporting that direct activation of endogenous TRPC channels in chromaffin cells can trigger exocytosis, this again suggests how AzPico may be used to exert functional control over endogenous biology (here, for optically controlled release of adrenaline).

### TRPC4/5 photoswitches are effective in tissue slices and can reveal channel-specific biology

**Fig. 5:**
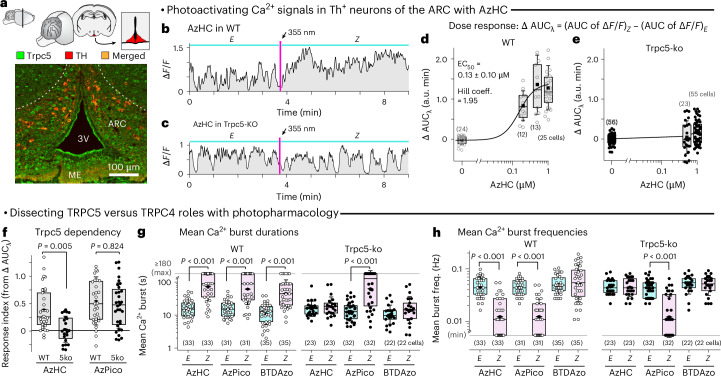
AzHC and AzPico are potent photoswitchable activators of TRPC-dependent Ca^2+^ responses in mouse hypothalamus. **a**, Coronal brain slice: cartoon with ARC region in red and microscopy image with Trpc5 immunostained in green and Th^+^ neurons in red (representative image from *n* = 4 mice). **b**–**h**, Ca^2+^ responses in Th^+^ neurons of Th-GCaMP6f (WT) or Th-GCaMP6f-ΔTrpc5 (Trpc5-ko or 5ko) mice. **b**,**c**, Single-cell Ca^2+^ traces before (*E*) and after (*Z*) a 355-nm pulse. **d**,**e**, ΔAUC_λ_, light dependency of the AUC as acquired in **b**,**c**. **f**, AzHC only chromocontrols TRPC5-dependent Ca^2+^ responses; AzPico can chromocontrol Ca^2+^ responses by another route (likely TRPC4). **g**,**h**, mean Ca^2+^ burst durations and frequencies in WT and 5ko upon *E*→*Z* photoswitching. AzHC/AzPico, 500 nM (except in dose–response study); BTDazo, 10 µM. In **d**–**h**, each point represents one cell with *n* cells per group. Box plots show the interquartile ranges, median (line), mean (black rhombus) and s.d. (whiskers). In **f**–**h**, statistical analysis was conducted using a Kruskal–Wallis analysis of variance (ANOVA) and Dunn’s *P* values are shown. In **g**,**h**, if no *P* value is indicated, then the data were not significantly different between the *E* and *Z* isomers of AzHC or BTDAzo (*P* = 0.052–0.999; **g**, Kruskal–Wallis ANOVA: *χ*^2^ (11) = 132.71744, *P* < 0.0001; **h**, Kruskal–Wallis ANOVA: *χ*^2^ (11) = 142.9224, *P* < 0.0001). Min–max values and all other details are provided in Supplementary Fig. 22 (full legend in Supplementary Information). Source data

We believed that AzHC and AzPico’s efficacy switch mechanism and high potency should make them effective chromocontrol reagents for deeper tissues and moved to test it. The hypothalamic arcuate nucleus (ARC) (Fig. 5a) is a signaling center in the brain where most dopamine (Th^+^) neurons are known to express TRPC5, which contributes not only to spontaneous oscillatory burst-firing activity and Ca^2+^ burst responses^34^ but also to sustained activation following stimulation with the hormone prolactin (a mechanism of reproductive signaling that has been conserved for >300 million years)^12,34^. The temporal distinction between these activation modes is striking and suggests the possibility of using either AzHC or AzPico as a time-resolved TRPC5 probe. However, we remain ignorant even of whether the most closely related congener TRPC4 has a role in this circuitry; hence, we were particularly drawn to apply these reagents comparatively, hoping that their intersecting C4/C5 selectivities could deliver new information about TRPC4 biology in endogenous systems.

We therefore took 275-µm-thick mouse brain slices through the dorsomedial ARC (Fig. 5a) and imaged the Ca^2+^ indicator GCaMP6f in Th^+^ neurons while photoswitching AzHC or AzPico to study TRPC[4]/5 photoactuation in situ. Ca^2+^ influx should be seen across several independent parameters: from time-resolved aspects such as longer Ca^2+^ burst durations and lower Ca^2+^ burst frequency to simply larger areas under the curve of the fluorescent Ca^2+^ signal (AUC), which translate into a ‘response index’ > 1 (details in Supplementary Fig. 22). Pleasingly, we again found that AzHC and AzPico can strongly photoswitch Ca^2+^ responses at endogenous TRPC levels by all these metrics. A single ≥23-ms pulse of 355-nm light (*E*→*Z*) induced dramatically sustained high-Ca^2+^ signals lasting up to ≥3 min (42% of cells; Fig. 5b,d,g,h). TRPC5 was sufficient for this signal, as TRPC5-knockout slices did not respond to AzHC photoswitching (Fig. 5c,e). However, *Z*-AzPico Ca^2+^ photoresponses were maintained despite TRPC5 knockout, providing functional evidence for a role of TRPC4 or TRPC1/C4 in the Ca^2+^ response in dorsomedial Th^+^ neurons of the ARC (Fig. 5f–h and Supplementary Fig. 22c,d), which can now be further explored^12^.

This discovery underscores the utility of this pair of photoswitches for deconvoluting the roles of TRPC4 from TRPC5. We also stress how important it was for these studies that the reagents were high-potency and ideal efficacy photoswitches. This allowed internally baselining signals after compound application (to overcome expression heterogeneity) and then photoswitching activity on from zero background at a precisely defined time in a fully reproducible wavelength-dependent manner (all needed for reliable statistics). Temporally modulated studies beyond the scope of this report are already underway, motivated by the xanthines’ sustained (low-frequency) Ca^2+^ bursts that contrast the photoswitchable TRPC5 activator BTDazo (burst frequency barely affected; Fig. 5h). This points to a rich interplay of pharmacology and spatiotemporally resolved biology in complex tissues, which photoswitchable reagents are uniquely poised to tackle.

### Photoswitching tissue-level physiology via TRPC4-based chromocontrol of intestinal motility

**Fig. 6:**
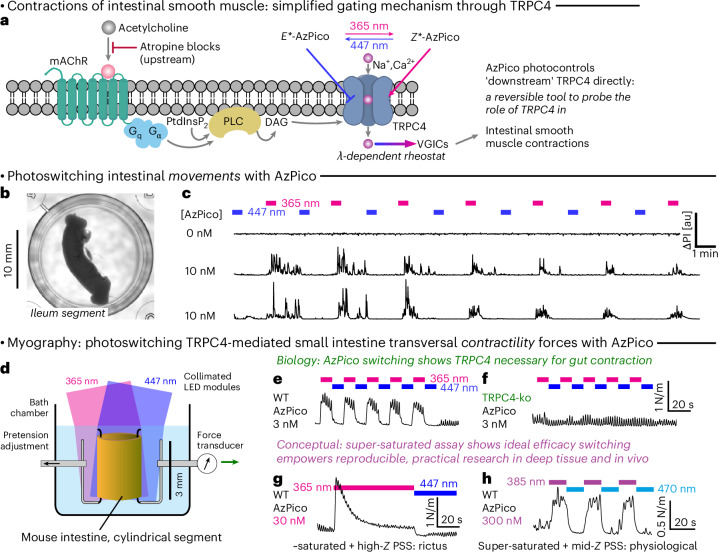
AzPico chromocontrol of intestinal contractility shows the key role of TRPC4 and power of ideal efficacy switching. **a**, Simplified molecular mechanism for TRPC4-dependent intestinal contractility^75^, which is directly testable using AzPico (E*/Z* indicate *E*/*Z-*rich-PSSs). **b**,**c**, Intestinal segments (mouse ileum) whose motility was blocked by atropine but treated with AzPico were driven into phases of fast macroscopic motions by UV photoswitching (longitudinal and ring muscle contractility) and then returned to immobility by blue light, reversibly over many cycles (Supplementary Video 5). **d–f**, Physiological-like ring contractility is reversibly stimulated and suppressed by alternating UV and blue illumination of segments treated with AzPico in a TRPC4-dependent manner. **g**,**h**, The ideal efficacy switch paradigm allows fully reproducible control of deep tissue bioactivity, by leveraging the saturation of dose and photon flux (hard to titrate) but the selection of wavelength (easy to choose) (Supplementary Fig. 23) (full legend in Supplementary Information). Source data

After observing chromocontrol in thin tissue slices by microscopy, we next tested for photoactuation of macroscopic downstream processes in thick tissue sections of the intestine. Landmark papers by Freichel^70^ and Zholos^16^ used irreversible suppression assays to suggest that TRPC4 activation, downstream from muscarinic acetylcholine receptors (mAChRs, the target of atropine), should be a critical component controlling small intestine peristalsis. Intestinal segment contractions are macroscopically coordinated oscillatory motions overlaid on a ‘tonic’ baseline contractile force. A subthreshold oscillatory pacemaker potential is amplified by mAChR through phospholipase C and TRPC4 activation to surpass the threshold potential of voltage-gated Ca^2+^ channels, leading to peristaltic contractions (Fig. 6a). We, therefore, expected that direct TRPC4 activation by *Z-*AzPico might hijack the pacemaker signal to drive oscillatory contractility, even with upstream signaling by mAChR blocked, and aimed to test this so as to directly elucidate the role of TRPC4 activation in intestinal contractility in endogenous tissues.

We took fresh 8–12-mm-long segments of mouse small intestine (jejuneal and ileal), added low atropine concentrations (300 nM) to paralyze their spontaneous motility and monitored their contractility by macroscopic video imaging while photoswitching AzPico (Fig. 6b). Indeed, even with just 10 nM AzPico, ultraviolet (UV) illumination initiated vigorous motions that were stopped rapidly by applying 447-nm light and then restarted with UV; the on–off chromocontrol could be cycled many times (Fig. 6c and Supplementary Video 5). No intestinal light responses were evident without AzPico and, highlighting the tissue-specificity involved, AzPico-treated colon segments also had no photoresponse.

We next analyzed intestinal contractility quantitatively, monitoring contractile muscle force in 3-mm-long ileal or jejuneal segments by myography during AzPico photoswitching with UV and blue light (Fig. 6d). With moderate AzPico concentrations (for example, 3 nM), 365-nm UV illumination not only increased tonic forces but also doubled oscillatory forces (1.9×) without changing oscillatory frequency, indicating that *Z-*AzPico ‘rides’ the circuit to drive typical contractility. Basal tension was then restored under 447-nm light and 365/447-nm cycling could be repeated many times (Fig. 6e). We assigned this intestinal chromocontrol to TRPC4 as segments of TRPC4-deficient mice^71^ never responded to *E*/*Z-*AzPico (Fig. 6f), while tonic and oscillatory force photoresponses in TRPC5-deficient mice were indistinguishable from those in matched control mice.

Our findings, thus, support that TRPC4 activation is both necessary and sufficient to convert subthreshold oscillatory signals into effective peristaltic motility in the small intestine. On an applied level, the AzPico-induced, optically tunable reactivation of atropine-paralyzed intestinal segments suggests potential experimental therapies to treat intestinal motility insufficiency conditions such as toxic or postoperative paralytic ileus. On a more conceptual level, the full and rapid photoreversibility of both gross motility and myographic readouts, assessed on the tissue level in WT samples, gives high confidence in the molecular TRPC4 specificity of intestinal contractility. We stress, however, that this simple and direct result is only possible for the ideal efficacy photoswitch, whereas classical high-affinity compounds such as EA or Pico145 practically cannot be washed out; hence, internally baselined, reversible experimentation without fatigue-based rundown or gross toxicity^72^ was previously impossible. We also highlight that we used a small-molecule photoswitch to elucidate a target through hypothesis-driven research, instead of creating and using a photoswitch after target identification.

However, given the in vivo potential of ideal efficacy photoswitches, we are certain that this will not be the last such case. A practical example of the value of chromocontrol that such photoswitches finally enable is illustrative. Our protocols converged to the use of 365 nm for high-completion *E*→*Z* switching and best channel activation; however, with moderate to high concentrations of AzPico (30 nM), this resulted in rictus-like intestinal tensioning and rundown, presumably through overstimulation (Fig. 6g). This seems to be a regular biological situation, whereby a desired, physiological effect is only reached when a target protein is partially but not fully stimulated. This overstimulation could be avoided in a classical way by titrating AzPico concentrations down to ~3 nM (Supplementary Information) but ideal efficacy switching should allow the much simpler workaround of merely changing the illumination wavelength. To test the ideal model in Fig. 1g–i as stringently as possible, we saturated tissues using 300 nM AzPico but adopted 385 nm instead for UV illumination (also applied as saturating illumination). This 20-nm wavelength shift perfectly and reproducibly drove only partial channel activation, giving the physiology-like oscillatory intestinal contractions we sought (Fig. 6h). We feel that using both saturating ligand concentration and saturating light fluence is key for avoiding the practical irreproducibilities of ‘tuned compound and light dosing’ that is required for affinity switches to operate and illustrates why a paradigm shift to ideal efficacy photoswitching can empower deep tissue or in vivo research, even for targets (for example, TRPC4/5) that are nonlinearly responsive and have time-dependent and dose-dependent bioactivity.

## Discussion

We rationally developed AzHC and AzPico as ideal efficacy photoswitches for TRPC[4]/5. Their photoisomerization flips them between *E* inverse agonists and *Z* agonists, with both forms having excellent binding affinity, resulting in a pair of lit-active low-nanomolar-potency tools that can be used together to elucidate TRPC5-selective or TRPC4-selective biology. To date, there have only been two reports of protein–ligand structures where both *E* and *Z* isomers of the same azobenzene reagent were bound^49,66^; we now report two more, with both TRPC4 and TRPC5 *E*/*Z* structure pairs, which will help progress design rules for efficacy switches on other targets. Unlike nearly all photopharmaceuticals, the bioactivity of AzHC and AzPico is fully determined by the illumination wavelength used but not their concentration. This makes them suitable for reliable and reproducible chromocontrol across diverse model systems, from overexpression in HEK cells to endogenous expression in primary neurons, adrenalin-secreting cells, brain slices and intact segments of intestine, where they elegantly support a hypothesis for TRPC4-dependent contractility.

Importantly for the TRPC field, the tissue-level chromocontrol by nanomolar AzPico and AzHC marks these reagents as exceptionally effective optical tools for precisely and reversibly manipulating endogenous TRPC biology^17^ in situ. Their likely applications, thus, stretch far beyond the muscular and neuronal applications shown here, toward elucidating TRPC4/5’s rich and largely still cryptic biology. Lastly, the rapid macroscopic photoswitchability of AzPico’s downstream secondary and tertiary effects (that is, not only ion flux but also integration in the native cascade controlling tissue-scale muscle movement) is highly unusual if not unique in photopharmacology (Fig. 6).

For biology and chemistry in general, we showed that the ideal efficacy photoswitching paradigm can be rationally chemically designed and rationally biologically exploited to achieve robust high-precision control of endogenous systems that is purely directed by light rather than drug dosing and distribution. This feature combination makes it suited excellently for deep tissue and in vivo work. We introduced some general biological target considerations and general chemical design concepts to ground the wider introduction of efficacy photoswitching (Supplementary Note 2). Of course, the pharmacological requirement to design an efficacy photoswitch is that an efficacy ‘cliff’ should be plausible and this is not the case for many families of highly active drugs. For example, substrate or cosubstrate mimics that inhibit enzymes in their active site cannot be made to instead agonize enzyme activity while still binding; thus, they are not convertible into efficacy photoswitches. By contrast, allosteric modulatory sites (which, in many cases, are already known to allow both positive and negative allosteric modulation) seem ideally feasible candidates from the pharmacological perspective. Furthermore, our perception is that this target-based design opportunity is closely matched to real, biologically based needs. We define ‘poised targets’ as being those proteins that need to rapidly and spatiotemporally modulate their functions to support life (ion channels, receptors, sensory or signaling integrators, etc.). We perceive that such poised targets are most likely to be addressable by the efficacy switch paradigm, as they are often evolved to allow small modulators or ligands to bias their activity. Moreover, we perceive that these are also the targets that most urgently require modulatory reagents delivering high spatiotemporal resolution across the scales even to in vivo settings (as efficacy photoswitch reagents promise) to understand their endogenous biology. That match of need to opportunity is not accidental; it simply reflects how biology has evolved and perfected its own ligand-based or protein-based regulatory mechanisms to operate in complex environments. Accordingly, efficacy photoswitching can be an ideal way to harness this potential to the fullest.

Lastly, we encourage chemists to take up awareness of this possibility, to rigorously test for it even where it was not a design aim^29,54,55^ and to work toward rationally using the efficacy photoswitching paradigm to generate a cornucopia of reagents that are more in vivo competent rather than remaining locked to affinity photoswitching approaches. We foresee that such efficacy photoswitches can unlock a new era for chromocontrolled biology, in ways that not only deeply impact chemical or cell culture proof-of-principle studies but translate seamlessly across to basic physiology and medical research, enabling researchers to noninvasively probe and modulate endogenous pathways in deep tissues and in vivo.

## Methods

Materials and methods including chemical synthesis, photophysical data and NMR spectra are available in the Supplementary Information.

## Data and material availability

All data needed to evaluate the conclusions in the paper are present in the paper and/or the Supplementary Information. Data were also deposited and are freely available on bioRxiv^73^ and figshare^74^. Cryo-EM structures were deposited to the Protein Data Bank and EM Data Bank under the following accession codes: TRPC4DR:*E*-AzPico, 3.0 Å, PDB 9FXL, EMD-50850; TRPC4DR:*Z*-AzPico, 3.1 Å, PDB 9FXM, EMD-50851; hTRPC5:*E*-AzHC, 2.6 Å, PDB 9G4Y, EMD-51074; hTRPC5:*Z*-AzHC, 2.9 Å, PDB 9G50, EMD-51076.

### Reporting summary

Further information on research design is available in the Nature Portfolio Reporting Summary linked to this article.

## Online content

Any methods, additional references, Nature Portfolio reporting summaries, source data, extended data, supplementary information, acknowledgements, peer review information; details of author contributions and competing interests; and statements of data and code availability are available at 10.1038/s41589-025-02085-x.

## Supplementary information

Supplementary Information Supplementary Notes 1–5, Figs. 1–40, Tables 1–7, Chemistry and Methods.

Reporting Summary

Supplementary Video 1 Video of 3D Cryo-EM structure of TRPC4 with *E*-AzPico bound.

Supplementary Video 2 Video of 3D Cryo-EM structure of TRPC4 with *Z*-AzPico bound.

Supplementary Video 3 Video of 3D Cryo-EM structure of TRPC5 with *E*-AzHC bound.

Supplementary Video 4 Video of 3D Cryo-EM structure of TRPC5 with *Z*-AzHC bound.

Supplementary Video 5 Video of photoswitching intestinal movement.

## Source data

Source Data Fig. 1 Statistical source data.

Source Data Fig. 2 Statistical source data.

Source Data Fig. 4 Statistical source data.

Source Data Fig. 5 Statistical source data.

Source Data Fig. 6 Statistical source data.
