# Supplementary material for: Ideal efficacy photoswitching for chromocontrol of TRPC4/5 channel functions in live tissues
Source: Nat Chem Biol. 2026 Jan 16;22(2):180–91. doi: 10.1038/s41589-025-02085-x (PMC12858405; doi:10.1038/s41589-025-02085-x)
Supplement: Supplementary file 2 — Reporting Summary [file 41589_2025_2085_MOESM2_ESM.pdf]

nature portfolio

Corresponding author(s): Oliver Thorn-Seshold, Michael Schaefer

Last updated by author(s): Oct 14, 2025

Reporting Summary

Nature Portfolio wishes to improve the reproducibility of the work that we publish. This form provides structure for consistency and transparency in reporting. For further information on Nature Portfolio policies, see our Editorial Policies and the Editorial Policy Checklist.

Statistics

For all statistical analyses, confirm that the following items are present in the figure legend, table legend, main text, or Methods section.

n/a	Confirmed	
☐	☒	The exact sample size (*n*) for each experimental group/condition, given as a discrete number and unit of measurement
☐	☒	A statement on whether measurements were taken from distinct samples or whether the same sample was measured repeatedly
☐	☒	The statistical test(s) used AND whether they are one- or two-sided<br>*Only common tests should be described solely by name; describe more complex techniques in the Methods section.*
☒	☐	A description of all covariates tested
☐	☒	A description of any assumptions or corrections, such as tests of normality and adjustment for multiple comparisons
☐	☒	A full description of the statistical parameters including central tendency (e.g. means) or other basic estimates (e.g. regression coefficient) AND variation (e.g. standard deviation) or associated estimates of uncertainty (e.g. confidence intervals)
☐	☒	For null hypothesis testing, the test statistic (e.g. *F*, *t*, *r*) with confidence intervals, effect sizes, degrees of freedom and *P* value noted<br>*Give P values as exact values whenever suitable.*
☒	☐	For Bayesian analysis, information on the choice of priors and Markov chain Monte Carlo settings
☒	☐	For hierarchical and complex designs, identification of the appropriate level for tests and full reporting of outcomes
☒	☐	Estimates of effect sizes (e.g. Cohen's *d*, Pearson's *r*), indicating how they were calculated

Our web collection on statistics for biologists contains articles on many of the points above.

Software and code

Policy information about availability of computer code

Data collection	As related to Figure 1: Figure 1k-n (and S2, S12 and S13): Fluo-4 fluorescence signals were recorded in a custom-made Fluorescence Imaging Plate 182 Reader (FLIPR) built into a robotic liquid handling station (Freedom Evo 150, Tecan, 183 Männedorf, Switzerland) using a Zyla sCMOS camera (Andor, 189 Belfast, UK) and the controlling software Micromanager (Edelstein et al., 2010). Figure 1c-d, S9-11: Absorption was measured with a Agilent Cary60 Spectrophotometer, irradiation carried out with a CoolLED pE-4000 and controlling software Micro-Manager.<br>As related to Figure 2: Electrophysiological experiments were performed at room temperature using a Multiclamp 700B amplifier with a Digidata 1440A digitizer (Axon CNS, Molecular Devices, Sunnyvale, CA) controlled with PClamp 10 software (Molecular Devices). Figure 2 h-j, Table S3: PSS data was collected on a HPLC (details see supporting information).<br>As related to Figure 3: TRPC5 structural biology: Cryo-EM data were collected using the standard EPU data collection software installed by FEI EPU 3.1. TRPC4 structural biology: Cryo-EM datasets were collected using the commercially available software EPU version 2.8<br>As related to Figure 4:Electrophysiological recordings were peformed using HEKA Pulse 8.2 software (Lambrecht, Germany); Image of TRPC5 IC R26 tGFP positive neuron was taken with VisiView software (Visitron, Germany).<br>As related to Figure 5: Data acquisition upright scanning confocal microscope (Zeiss LSM 880 Indimo) equipped with a standard Argon laser for GCAMP6f excitation at a wavelength of 488 nm and a UV laser (Coherent) emitting 355 nm for photoswitching; Zen<br>As related to Figure 6: Figure 6b,c, Figure S23b, and Video S5: Optical recordings of intestinal motility were obtained with a Zyla sCMOS camera (Andor, Belfast, UK) and the controlling software Micromanager (Edelstein et al., 2010). Figure 6e-h and Figure S23c-g: Contractile forces were measured and digitized with a 4-channel multi myograph system (DMT 620M, Danish Myo Technology, Hinnerup, Denmark), controlled with aLabChart Pro V8 software.<br>As related to Figure S8: Intracellular calcium measurements used the SoftMax Pro 7 software.
Data analysis	As related to Figure 1k-n (S2a-d, S12 and S13): Micromanager/Fiji; c-d, S9-11: GraphPad Prism 10

Data analysis

As related to Figure 2: Clampfit 10.7, h-j: GraphPad Prism 10 and Agilent ChemStation
As related to Figure 3: TRPC5 structural biology: Data was pre-process (Motion correction and CTF estimation) in CryoSPARC v4.1 while particle picking, and data processing was done in Cryosparc v4.4. Initial model was generated using ModelAngelo, and manually adjusted in Coot (0.9.8.8) while for model refinement we used PHENIX (1.20.1). Model validation, as reported in the paper was conducted using PHENIX and Molprobity. UCSF ChimeraX (1.6) was used for visualisation and figure preparation. TRPC4 structural biology: Data collection was monitored live using TranSPHIRE. Intial motion correction and dose weighting was done with MotionCor2 v.1.3.0 and CTF estimation with CTFFIND 4.1.131. SPHIRE software package version 1.4 was used for Particle extraction and 2D classification. Particles were picked automatically with crYOLO version 1.8. 3D classification and refinement was performed with Relion v 3.1 and CryoSPARC v 4.0. Protein model building was carried out in coot v 1.9 and further refined by Phenix v 1.18.2. Figures were prepared in Chimera v 1.15 and Chimera X v 1.7.1. Pore analysis has been done by Pore Walker online Software.
As related to Figure 4: Data was analysed using IgorPro 5.0 and SigmaPlot 13.
As related to Figure 5: ImageJ, Igor Pro (Wavematrics), OriginLab v. 2020.
As related to Figure 6: Figure 6b,c: MicroManager 2.0 gamma and MS Excel. Figure 6e-h: LabChart Pro V8 (ADInstruments) and MS Excel
As related to Figure S8: Analysis of data from intracellular calcium measurements was performed using GraphPad Prism 8, 9, 10

For manuscripts utilizing custom algorithms or software that are central to the research but not yet described in published literature, software must be made available to editors and reviewers. We strongly encourage code deposition in a community repository (e.g. GitHub). See the Nature Portfolio guidelines for submitting code & software for further information.

Data

Policy information about availability of data

All manuscripts must include a data availability statement. This statement should provide the following information, where applicable:

- Accession codes, unique identifiers, or web links for publicly available datasets
- A description of any restrictions on data availability
- For clinical datasets or third party data, please ensure that the statement adheres to our policy

All data needed to evaluate the conclusions in the paper are present in the paper. Data are deposited in BioRxiv (https://doi.org/10.1101/2024.07.12.602451), or on Figshare (Fig. 5, https://doi.org/10.6084/m9.figshare.26232254.v1), or on PDB and EMDB as related to Figure 3 (TRPC4:E-AzPico, 3.0 Å, PDB 9FXL (https://doi.org/10.2210/pdb9FXL/pdb), EMDB 50850; TRPC4:Z-AzPico, 3.1 Å, PDB 9FXM (https://doi.org/10.2210/pdb9FXM/pdb), EMDB 50851; hTRPC5:E-AzHC, 2.6 Å, PDB 9G4Y (https://doi.org/10.2210/pdb9G4Y/pdb), EMDB 51074; hTRPC5:Z-AzHC, 2.9 Å, PDB 9G50 (https://doi.org/10.2210/pdb9G50/pdb), EMDB 51076).

Research involving human participants, their data, or biological material

Policy information about studies with human participants or human data. See also policy information about sex, gender (identity/presentation), and sexual orientation and race, ethnicity and racism.

Reporting on sex and gender	n/a
Reporting on race, ethnicity, or other socially relevant groupings	n/a
Population characteristics	n/a
Recruitment	n/a
Ethics oversight	n/a

Note that full information on the approval of the study protocol must also be provided in the manuscript.

Field-specific reporting

Please select the one below that is the best fit for your research. If you are not sure, read the appropriate sections before making your selection.

☒ Life sciences ☐ Behavioural & social sciences ☐ Ecological, evolutionary & environmental sciences

For a reference copy of the document with all sections, see nature.com/documents/nr-reporting-summary-flat.pdf

Life sciences study design

All studies must disclose on these points even when the disclosure is negative.

Sample size

As related to Figure 1k-n (S2, S12, S13): A cell suspension was used and all experiments were conducted as a minimum of 3 independent experiments with each experiment averaging data in technical duplicates.
As related to Figure 2d-g: Data as means and S.E. were obtained from 12 different cells, in our hand a sufficient number to estimate electrophysiological activity of a compound when using a clonal HEK293 cell line.
As related to Figure 3: Structural biology of TRPC4: For AzPico E isomer, 2,598 movies were collected. 214,819 particles were picked and for final reconstruction and 139,750 particles were used; For AzPico Z isomer 3,311 movies were collected. 232983 particles were picked and for final reconstruction and 93651 particles were used.
As related to Figure 4: This experimental design for electrophysiological recordings is based on years of research experience conducting these

types of experiments (e.g. Schwarz et al., 2019; Kollewe et al., 2022). Statistical analysis was conducted on the collected data, taking into account the number of cells in each experiment. No specific statistical tools were used to predetermine the sample size. The sample sizes were chosen to provide sufficient statistical power to detect biologically significant differences between experimental treatments. All key experiments supporting the main conclusions of the study were repeated several times in samples prepared from at least 3 animals obtained from different litters.
As related to Figure 5: Sample sizes are estimated based on previous physiological studies using similar animal models to guarantee statistical relevance. The number of animals used is a minimum necessary to provide adequate data to test the hypotheses of this project. We have minimized the number of animals required by the animal welfare committees wherever possible. PHYSIOLOGY: Tissue from a single animal remains maximally viable for a full day's experiments. However, due to animal viability, cell viability, and experimental variables it is impossible to predict how many responding cells can be successfully recorded in a given day's work. The significance of a particular recording, and hence the judgment as to whether it must be repeated, can only be made after the recordings are analyzed. Some experiments required tissue from 3-5 different animals. Any less data would fail to meet statistical significance.
As related to Figure 6: As related to Figure 6b and Figure S23b: optical recordings of intestinal motility were obtained from 10 intestinal segments obtained from a single C57Bl6/N mouse.

As related to Figure 6e-h and Figure S23c-g: Data are representative traces obtained from 2-4 intestinal segments per animal. Segments from four 7- to 10-week-old male (TRPC5-/-) and five (TRPC4-/-) mice were investigated for each genotype and genetic background (TRPC4-/- mice, matching wild-type mice, TRPC5-/- mice, and their matching wild-type.
As related to Figure S8: intracellular calcium recordings were used to show that the point mutants generated led to TRPC channels that retained sensitivity to (-)-englerin A but did not necessarily retain sensitivity to AM237 / Z-AzHC. Sample size calculations were not considered applicable to these studies. The number of repeats was deemed sufficient to support the conclusions drawn.

Data exclusions

As related to Figure 1: There was no data excluded.
As related to Figure 2d-g: Using a clonal HEK293 cell line stably expressing YFP-tagged mTRPC4ß enabled the selection of mTRPC4-positive cells by fluorescence signal and avoided a strong mean variation. Only 1 measurement of 12 cells was excluded from statistical analysis because maximal activity was too small to obtain a realistic action spectra.
As related to Figure 3: TRPC5 structural biology: No data were excluded. TRPC4: During cryo-EM dataset processing, false picks during particle picking were eliminated and further particles that does not contribute to high resolution features has been removed based on 2D and 3D classification which is a standard procedure.
As related to Figure 4: There was no data excluded.
As related to Figure 5: Recordings were excluded in the determination of the dose-response curves, when a cell was not responding to any stimulation. Otherwise no data were excluded.
As related to Figure 6: There was no data excluded.
As related to Figure S8: At most, one outlier well per plate was excluded e.g. in the case that transfection had not succeeded in that well. Independent replicates are not shown.

Replication

As related to Figure 1k-n (S2, S12, S13): Reproducibility was was ascertained by performing measurements on 3 several days, with technical duplicates.
As related to Figure 2d-g: Reproducibility of electrophysiological results was ascertained by performing measurements on several days.
As related to Figure 3: TRPC5 structural biology:N/A; no clinical samples or animal samples were used. Randomization is used CryoSPARC to estimate the final resolution of cryo-EM maps. TRPC4: All cryo-EM datasets were acquired once as it is unattainable to repeat the cryo-EM dataset collection and processing of the same sample from the time and cost perspective.
As related to Figure 4: All experiments were performed from at least three independent preparations and measurements.
As related to Figure 5: Multiple measurements using different mice in each experimental and control groups were performed to check reproducibility of results. As has been our practice throughout the manuscript, we use at least three animals per experiment.
As related to Figure 6: 10-12 repeated photoswitching cycles were applied in parallel measurements of 2-4 intestinal segments. Experiments were repeated at three different days, using segments obtained from different animals
As related to Figure S8: Total repeats (N) are shown in Figure S4. Independent replicates are not shown.

Randomization

As related to Figure 3: During final cryo-EM dataset reconstruction particles were randomly split into two equal subsets for FSC calculation.
As related to Figure 4: Wildtype and corresponding knock out cells were prepared and measured on the same day in a random fashion.
As related to Figure 5: To eliminate the potential influence of batches of physiological solutions or solutions of photoswitchable compounds, the experiments with wild-type and knockout Trpc5 animals were conducted on the same day. Furthermore, changes in the concentration of photoswitchable compounds were randomized to exclude adaptive responses or mistakes in washing out a particular concentration from the external solution.

Blinding

Investigators were not blinded for this study (not relevant to cellular or ex vivo studies with automated objective readout by machines)

Reporting for specific materials, systems and methods

We require information from authors about some types of materials, experimental systems and methods used in many studies. Here, indicate whether each material, system or method listed is relevant to your study. If you are not sure if a list item applies to your research, read the appropriate section before selecting a response.

Materials & experimental systems

n/a	Involved in the study
☐	☒ Antibodies
☐	☒ Eukaryotic cell lines
☒	☐ Palaeontology and archaeology
☐	☒ Animals and other organisms
☒	☐ Clinical data
☒	☐ Dual use research of concern
☒	☐ Plants

Methods

n/a	Involved in the study
☒	☐ ChIP-seq
☒	☐ Flow cytometry
☒	☐ MRI-based neuroimaging

Antibodies

Antibodies used

rabbit anti-Th (1:1000, polyclonal, #ab112, Abcam; RRID:AB_297840) ; donkey anti-rabbit-Alexa555 (1:1000, #A31572, Thermo Fisher; RRID:AB_162543); unconjugated AffiniPure Fab fragment goat anti-mouse IgG (H+L; #115-007-003, Jackson ImmunoResearch; RRID:AB_2338476); mouse anti-Trpc5 (1:500, monoclonal; clone N67/15, NeuroMab, Davis, CA; RRID:AB_2240979); biotinylated goat anti-mouse IgG2b antibody (1:400, #115-065-207, Jackson Immuno Research; RRID:AB_2338573); Alexa 488-conjugated streptavidin (1:200; S-32354, Invitrogen; RRID:AB_2315383)

Validation

For negative controls, antibody dilution buffer without primary antibodies was applied. The exact same batch of mouse anti-Trpc5 (1:500, monoclonal; clone N67/15, NeuroMab, Davis, CA; RRID:AB_2240979) antibody was investigated in TRPC5-deficient mice (Blum et al., 2019, Proceedings of the National Academy of Science USA).

Eukaryotic cell lines

Policy information about cell lines and Sex and Gender in Research

Cell line source(s)

As related to Figures 1-2: Human embryonic kidney (HEK293) cell line (ATCC CRL-1573); stably transfected with cDNA of mouse TRPC4ß-YFP or TRPC5-YFP.
As related to Figure 3: TRPC5 structural biology: Freestyle 293F (ThermoFisher Scientific, R79007).
As related to Figure S8: HEK293 (ATCC CRL-1573).

Authentication

We did not authenticate the HEK293 cell lines.

Mycoplasma contamination

We did not test for mycoplasma contamination.

Commonly misidentified lines
(See ICLAC register)

No commonly misidentified cell lines were used in this study.

Animals and other research organisms

Policy information about studies involving animals; ARRIVE guidelines recommended for reporting animal research, and Sex and Gender in Research

Laboratory animals

As related to Figure 4: TRPC1/C4/C5 triple knock out, TRPC5 single knock out mice and TRPC5 IC eR26 tGFP were generated as described previously (Bröker-Lai et a., 2017; Schwarz et al., 2019; Kollewe et al., 2022). Trpc1/4/5–/– and TRPC50/- mice were compared to C57BL/6N mice, which were obtained from Charles River and housed under the same conditions as the knockout (KO) animals. For hippocampal neuron preparations, animals of either sex were prepared postnatal day 0. Mouse chromaffin cells were prepared from adult male mice (10-12 weeks old)
As related to Figure 5: We used following mouse strains: 1. Th-GCaMP6f mice (produced by crossing mouse strain 'Th-Cre mice', also called B6.Cg-7630403G23RikTg(Th-cre)1Tmd/J (RRID:IMSR_JAX:008601 with mouse strain R26-GCaMP6f, also called B6;129S-Gt(ROSA)26Sortm95.1(CAG-GCaMP6f)Hze/J (RRID:IMSR_JAX:024105, ror Ai95D mice). 2. Th-GCaMP6f-ΔTrpc5 (produced by crossing Th-GCaMP6f mice with Trpc5-E5–/– mice (Trpc5tm1.1Lbi (RRID:IMSR_JAX:024535; MMRRC Stock No: 37349-JAX). All mice were Adult female mice (7 - 20 weeks old).
As related to Figure 6: mus musculus, C57Bl6/N strain (CharlesRiver) wild-type or homozygous TRPC4-/- and homozygous TRPC5-/-, backcrossed to the C57Bl6/N strain (CharlesRiver) for at least seven generations. Mice were housed in groups of two to three animals under approved standard conditions of a 12 h light-dark-regime and access to food and water ad libitum. Strains for testing intestinal contractility were C57BL6/N mice (from Charles River) and homozygous TRPC5 or TRPC4 single knockout mice(Freichel et al. Nat. Cell Biol. (2001) 3:121-127 PMID: 11175743), both back-crossed on the C57BL6/N background for at least five generations. All mice were of male sex. For myography experiments, intestinal segments were prepared from mice at the age of 4-7 months. Wild-type control mice were matched in age to the respective knock-out animals.

Wild animals

The study did not involve wild animals.

Reporting on sex

As related to Figure 4: For hippocampal neuron preparations, animals of either sex were prepared postnatal day 0. Only adult male mice were taken for chromaffin cell preparations. To avoid hormonal differences in adult mice influencing the response, we specifically chose only male mice.
As related to Figure 5: From the study of Blum et al. (2019) PNAS, it is evident that only female mice having a Trpc5 deficiency result

in hypoprolactinemia and altered function of oscillatory TH+ neurons in the arcuate nucleus. This is the rationale behind the decision to utilize only female mice in the experiments of Figure 5 and S22.
As related to Figure 6: In myography experiments, a total of 18 male mice (9 wild-type, 5 TRPC4-/- and 4 TRPC5-/- mice) have been investigated. Since expression of TRPC4 in the intestine is not reported to differ between male and female mice, we do not expect relevant differences between sexes.

Field-collected samples	The study did not involve samples collected from the field.
Ethics oversight	As related to Figure 4: All mice were kept according European Animal Welfare regulations and ethical guidelines from the local governing body (Nr Az. 2.4.1.3/ Bruns).<br>As related to Figure 5: Animal care and experimental procedures were performed in accordance with the guidelines established by the German Animal Welfare Act, European Communities Council Directive 2010/63/EU, the institutional ethical and animal welfare guidelines of the Saarland University (approval number of the Institutional Animal Care and Use Committee: CIPMM-2.2.4.1.1).<br>As related to figure 6: The study does not involve animal experiments. All tissues were explanted from animals that were previously sacrificed by decapitation.

Note that full information on the approval of the study protocol must also be provided in the manuscript.

Plants

Seed stocks	n/a
Novel plant genotypes	n/a
Authentication	n/a
